# Parabolic-accelerating vector waves

**DOI:** 10.1515/nanoph-2021-0255

**Published:** 2021-08-17

**Authors:** Bo Zhao, Valeria Rodríguez-Fajardo, Xiao-Bo Hu, Raul I. Hernandez-Aranda, Benjamin Perez-Garcia, Carmelo Rosales-Guzmán

**Affiliations:** Wang Da-Heng Collaborative Innovation Center, Heilongjiang Provincial Key Laboratory of Quantum Manipulation and Control, Harbin University of Science and Technology, Harbin 150080, China; School of Physics, University of the Witwatersrand, Private Bag 3, Johannesburg 2050, South Africa; Photonics and Mathematical Optics Group, Tecnologico de Monterrey, Monterrey 64849, Mexico; Centro de Investigaciones en Óptica, A.C., Loma del Bosque 115, Colonia Lomas del campestre, 37150 León, Gto., Mexico

**Keywords:** accelerating waves, structured light, vector beams

## Abstract

Complex vector light fields have become a topic of late due to their exotic features, such as their non-homogeneous transverse polarisation distributions and the non-separable coupling between their spatial and polarisation degrees of freedom (DoF). In general, vector beams propagate in free space along straight lines, being the Airy-vector vortex beams the only known exception. Here, we introduce a new family of vector beams that exhibit novel properties that have not been observed before, such as their ability to freely accelerate along parabolic trajectories. In addition, their transverse polarisation distribution only contains polarisation states oriented at exactly the same angle but with different ellipticity. We anticipate that these novel vector beams might not only find applications in fields such as optical manipulation, microscopy or laser material processing but also extend to others.

## 1 Introduction

The ingrained notion that light travels along a straight line was apparently defied in 2007 by Siviloglou et al. [1], who introduced a novel kind of light beam with the ability to self-accelerate along a parabolic trajectory upon free space propagation [2]. Such light beams, known as Airy beams, are natural solutions of the normalised paraxial wave equation (PWE). Crucially, even though they seem to propagate along parabolic trajectories, their first-order moment of the intensity (intensity centroid) propagates along straight lines, in accordance with the electromagnetic momentum conservation law. Along with the discovery of Airy beams, their fascinating properties prompted the development of novel applications, which have impacted a wide diversity of fields, such as, optical manipulation, microscopy, laser material processing, among others (see for example [3] for an extensive review). More importantly, the experimental demonstration of Airy beams ignited the quest for other kinds of accelerating beams [4–11]. Of particular interest is the case of accelerating parabolic beams, which form a complete and infinite orthogonal family of solutions of the normalised PWE [4, 12]. Such beams also propagate in free space in a non-diffracting way describing parabolic trajectories.

Noteworthy, most of the work carried out with accelerating beams has only considered the case of homogeneously polarised beams, while the manipulation of other degrees of freedom (DoF) is gaining popularity, giving rise to a more general class of beams generally known as structured light fields. This is the case of complex vector light beams, classically entangled in their spatial and polarisation DoF, which feature a non-homogeneous polarisation distribution across the transverse plane [13–15]. Such beams have gained popularity in recent times not only due to their unique traits such as their quantum-like non-separability [16–20] but also due to the many applications they are pioneering [21–27]. In vector beams, their spatial and polarisation DoF are coupled in a non-separable way, which generates the non-homogeneous polarisation distribution. Importantly, while the polarisation DoF is restricted to a two-dimensional space, the spatial one is not, as any of the unbounded solution sets of the wave equation, either in its exact or paraxial version, can be used. Examples of vector beams that have been experimentally demonstrated are Bessel, Laguerre–, Ince– and Mathieu–Gauss beams, amongst others, all of which propagate along straight trajectories [28–32]. Along this line, previous works have demonstrated the acceleration of vectorial fields, in which case, their polarisation structures rotate around the optical axis, while still propagate along straight lines [33]. Notably, only a few examples of vector beams propagating along non-straight trajectories are known so far [34, 35], being the Airy-vortex vector beam the only case with a parabolic trajectory [35].

We propose and experimentally demonstrate a new family of vector beams, which we term accelerating vector waves (AVWs), that are non-separable weighted superpositions of the polarisation and spatial DoF encoded in the orthogonal set of accelerating waves. These beams exhibit two interesting properties, namely, that their non-homogeneous polarisation distributions propagate in free space along parabolic trajectories maintaining a maximum degree of coupling, and that, even though the non-homogeneous transverse polarisation distribution of an individual AVW contains different states of elliptical polarisation, all of them are located on a great circle on the Poincaré sphere representation for polarisation. It is important to note that, although Airy and parabolic beams possess the same unusual accelerating properties, the parabolic beams have an inherent parabolic geometry, furthermore their intensity distributions are more localized than those of Airy beams [36]. This property also extends to AVWs, which makes them more suitable for practical applications, such as optical trapping and laser material processing. Here, we start by describing these beams theoretically, then move to their implementation in the laboratory, and finally show experimental results to showcase their novel features. Due to their intriguing properties, we expect AVWs will attract the wide interest of the optical community, stemming not only from their potential applications but also from their fundamental aspects.

## 2 Theory

**Figure 1:**
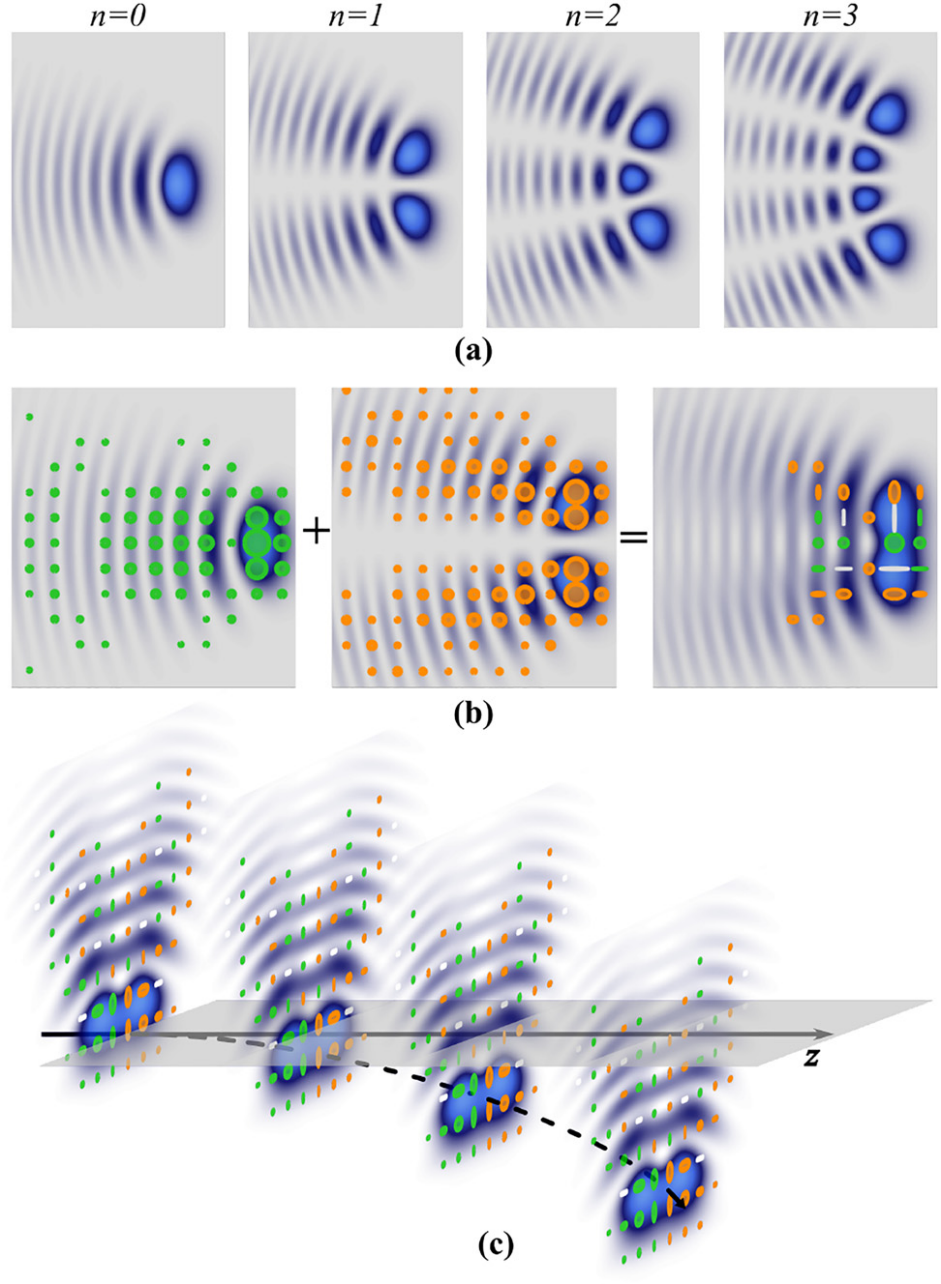
(a) Intensity profiles of accelerating parabolic scalar waves of orders *n* = {0, 1, 2, 3} at *z* = 0 mm. (b) Schematic representation of the non-separable superposition of two orthogonal scalar modes carrying orthogonal polarisations to generate an accelerating vector wave **AVW**_0,1_(*η*, *ξ*, *z*). (c) Conceptual illustration of the free-space propagation trajectory along the *z*-axis of the same mode shown in (b) (distances are not in scale). Green and orange ellipses represent right-handed circular polarisation (RHCP) and left-handed circular polarisation (LHCP), respectively, and white lines represent linear polarisation.

Accelerating parabolic waves (APWs) are solutions of the PWE in parabolic coordinates. In general, the conditions that a solution of the PWE have to satisfy to be an accelerating beam, have been studied by Bandres in [4, 5]. APWs are non-diffracting beams that accelerate during free-space propagation. Their experimentally realisable finite-energy form is given by [4]

(1) $$\begin{array}{ll} \phi_{n}\left(\eta ,\xi ,z\right)= & \exp\left[\frac{i}{2}\left(\frac{z}{2k\kappa^{2}}-ia\right)\left(\eta^{2}-\xi^{2}\right)\right]\\ & \times \exp\left[\frac{i}{3}{\left(\frac{z}{2k\kappa^{2}}-ia\right)}^{3}\right]\Theta_{n}\left(\eta \right)\Theta_{n}\left(i\xi \right), \end{array}$$

where the parabolic coordinates (*η*, *ξ*) are related to the Cartesian coordinates by $\left(\eta^{2}/2-\xi^{2}/2,\eta \xi \right)=\left\{x/\kappa -{\left[z/\left(2k\kappa^{2}\right)\right]}^{2}+iaz/\left(k\kappa^{2}\right),y/\kappa \right\}$ , *κ* is a transverse scale parameter, *k* is the wave number, *z* is the propagation distance and *a* is parameter that controls the exponential aperture of the beam at *z* = 0. The functions Θ_*n*_(⋅) correspond to the solutions of the differential equation

(2) $$\left(-\frac{1}{2}\frac{{\text{d}}^{2}}{\text{d}\eta^{2}}+\frac{\eta^{4}}{4}\right)N\left(\eta \right)=EN\left(\eta \right),$$

which corresponds to the one-dimensional (1D) Schrödinger equation with potential *V*(*η*) = *η* ^4^/4 (known as quartic potential) and *m* = *ℏ* = 1 [37]. Importantly, the eigen-solutions Θ_*n*_ ( n∈N ) of Eq. (2) form an orthogonal set of functions, whose parity is governed by *n*. Since these eigen-solutions cannot be expressed in a closed form, a suitable numerical method must be employed to obtain them [38]. In particular, we are interested in square integrable eigen-solutions of Eq. (2). Figure 1a shows the intensity profiles of the scalar APWs for *n* = {0, 1, 2, 3}. As can be seen, for *n* = 0 the intensity profile of the beam contains only one main lobe of maximum intensity and additional subsequent lobes of decaying intensity. In general, for *n* > 0 the intensity profile is formed by *n* + 1 lobes of maximum intensity.

Mathematically, the AVWs are constructed as a superposition of two scalar APWs with orthogonal polarisations, such that at the *z*-plane and for indices *n*, *m* they are given by

(3) $$\begin{array}{ll} {AVW}_{n,m}\left(\eta ,\xi ,z\right)= & \cos\;\alpha \;\phi_{n}\left(\eta ,\xi ,z\right){\hat{e}}_{1}\left(\theta ,\varphi \right)\\ & +\sin\;\alpha \;\exp\left(i\beta \right)\;\phi_{m}\left(\eta ,\xi ,z\right){\hat{e}}_{2}\left(\theta ,\varphi \right), \end{array}$$

where the weighting factor *α* ∈ [0, *π*/2] allows the balancing of the power associated with each orthogonal component and also enables the field to change from scalar (homogeneously polarised) to vector (non-homogeneously polarised). The parameter *β* ∈ [0, *π*] represents the inter-modal phase, in other words, *β* is the phase difference between the two orthogonal components and for simplicity is assigned to the second one. This parameter allows us to control the inter-modal phase. The basis vectors

(4) $${\hat{e}}_{1}\left(\theta ,\varphi \right)=\cos\left(\theta /2\right){\hat{e}}_{\text{R}}+\exp\left(i\varphi \right)\sin\left(\theta /2\right){\hat{e}}_{\text{L}},$$

(5) $${\hat{e}}_{2}\left(\theta ,\varphi \right)=\sin\left(\theta /2\right){\hat{e}}_{\text{R}}-\exp\left(i\varphi \right)\cos\left(\theta /2\right){\hat{e}}_{\text{L}},$$

represent the general elliptical polarisation basis. Note that we can obtain the LHCP/RHCP basis by setting *θ* = *π* and *φ* = 0 and the horizontal/vertical basis with *θ* = *π*/2 and *φ* = 0. Without loss of generality, here we will restrict our results to the circular polarisation basis, only briefly mentioning some theoretical examples of the horizontal/vertical basis. Figure 1b illustrates conceptually the above description for the specific case **AVW**_0,1_(*η*, *ξ*, *z*) with *α* = *π*/4 and *β* = 0 as polarisation distributions overlay onto their corresponding intensity profiles. Left and middle panels show the two scalar modes $\phi_{0}\left(\eta ,\xi ,z\right){\hat{e}}_{\text{L}}$ and $\phi_{1}\left(\eta ,\xi ,z\right){\hat{e}}_{\text{R}}$ with right circular polarisation and left circular polarisation, respectively, represented by green and orange ellipses for the first and second case respectively. Notice the intensity patters of the scalar modes are different, as required to obtain vector modes. In a similar way, the right panel presents the non-separable superposition of both scalar modes (Eq. (3)). The parabolic trajectory described by AVWs can be seen schematically in Figure 1c for **AVW**_2,3_(*η*, *ξ*, *z*) propagating along the *z* axis. Mathematically, this is expressed in the transverse shift *y*_s_ = [*z*/(2*k*)]^2^/*κ* ^3^ [36], which is independent of the indices *n* and *m*.

## 3 Experimental details

**Figure 2:**
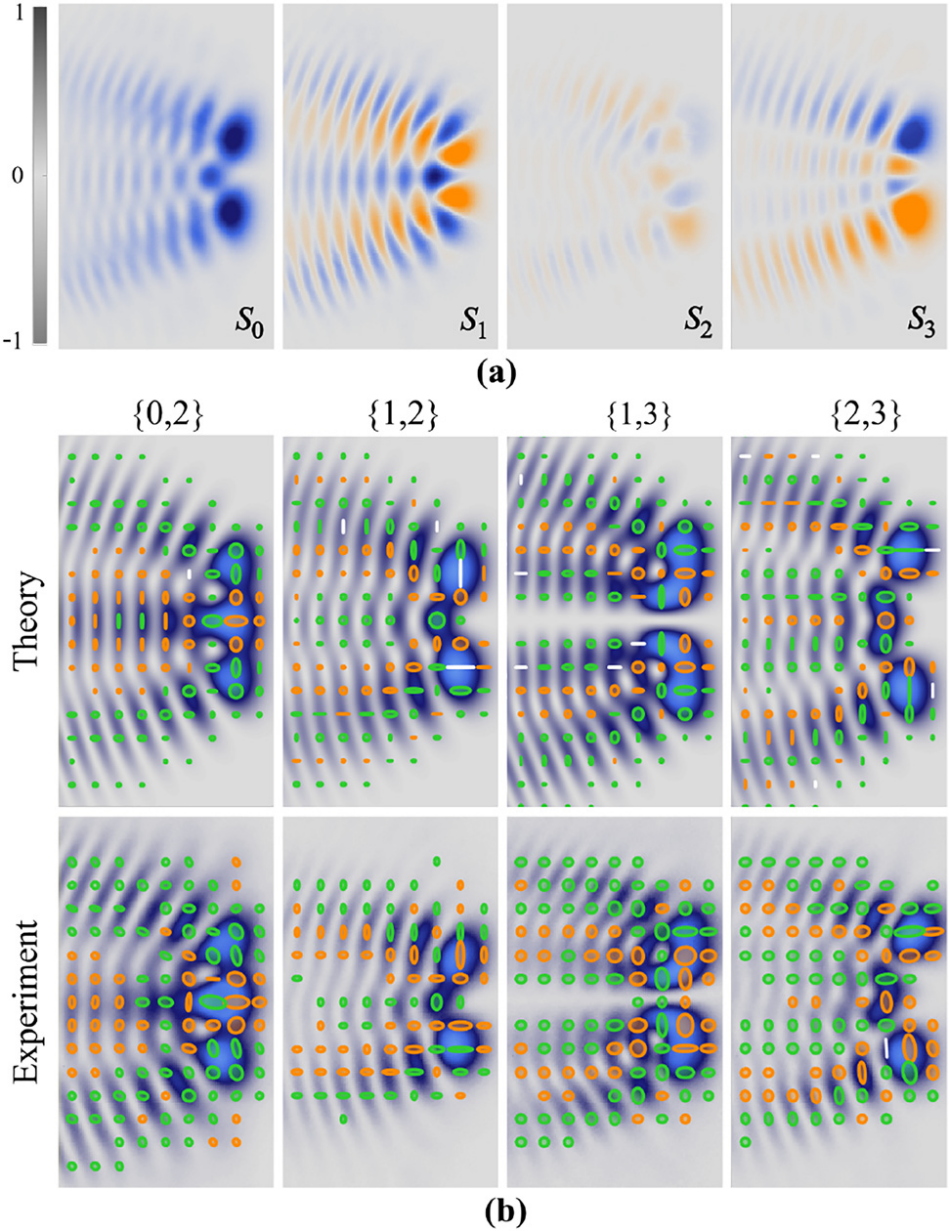
(a) Example of experimentally measured Stokes parameters *S*_0_, *S*_1_, *S*_2_ and *S*_3_ for **AVW**_1,2_(*η*, *ξ*, *z* = 0). (b) The theoretical and experimental reconstructed intensity and polarisation distribution from the Stokes parameters for the modes **AVW**_0,2_(*η*, *ξ*, 0), **AVW**_1,2_(*η*, *ξ*, 0), **AVW**_1,3_(*η*, *ξ*, 0) and **AVW**_2,3_(*η*, *ξ*, 0).

We implemented the AVW described above using a digital micromirror device (DMD) and following the technique that we proposed and fully characterised in a previous article [39]. This device is polarisation-insensitive, very flexible and versatile, allowing the generation of vector modes with arbitrary spatial distributions, such as elliptical or parabolic [31, 32, 34, 39]. In essence, a DMD is illuminated with two modes carrying orthogonal polarisations, impinging at slightly different angles but exactly at the geometric centre of the hologram displayed on the DMD. The hologram contains a superposition of the two transmittance functions that generate the constituting scalar modes of Eq. (3), each with an additional unique linear spatial grating that redirects the mode along a specific angle, and whose periods are carefully chosen to guarantee both generated beams co-propagate along the same axis, where the desired vector beam is created. Transmittance functions are calculated as the inverse Fourier transform of the desired modes *ϕ*_*n*_(*η*, *ξ*, *z*) [12, 36], thus we add a lens in a 2*f* configuration, where *f* is the focal length of the lens (*f* = 200 mm in our case) and measure at its back focal plane. Intensity patterns of the generated beams were captured with a high-resolution charge-coupled device (CCD) camera (FL3-U3-120S3C-C with a resolution of 4000 × 3000 pixels and a pixel size of 1.55 μm). Polarisation reconstruction was achieved through Stokes polarimetry, using a set of intensity measurements as detailed [32]

$$\begin{array}{ll} S_{0} & =I_{0},\\ S_{1} & =2I_{\text{H}}-S_{0},\\ S_{2} & =2I_{\text{D}}-S_{0},\\ S_{3} & =2I_{\text{R}}-S_{0}, \end{array}$$

where *I*_0_ is the total intensity, *I*_H_, *I*_D_ and *I*_R_ are the intensities of the horizontal, diagonal and right-handed polarisation components, respectively, and *S*_*i*_ (*i* = 0, 1, 2, 3) are the Stokes parameters. Figure 2a shows an example of the experimentally measured Stokes parameters *S*_0_, *S*_1_, *S*_2_ and *S*_3_ for the specific mode **AVW**_1,2_(*η*, *ξ*, *z* = 0). The reconstructed intensity and polarisation distributions of a set of representative examples of the experimentally generated **AVW**_*n*,*m*_(*η*, *ξ*, *z* = 0) modes using the circular polarisation basis are presented in Figure 2b, both for the theory (top row) and the experiment (bottom row). Notice that simulated and experimental results show high qualitative similarity both in the intensity and polarisation distributions, demonstrating the good performance of our generation method. However, it is important to mention the fact that differences in the polarisation distributions arise due to the high sensitivity of polarisation to small phase differences between the orthogonal components, which may arise experimentally due to different factors such as the non-flatness of the DMD screen, as well as other phase variations coming from the focussing elements and wave plates, some of which can be corrected as detailed in [40].

## 4 Results and discussion

**Figure 3:**
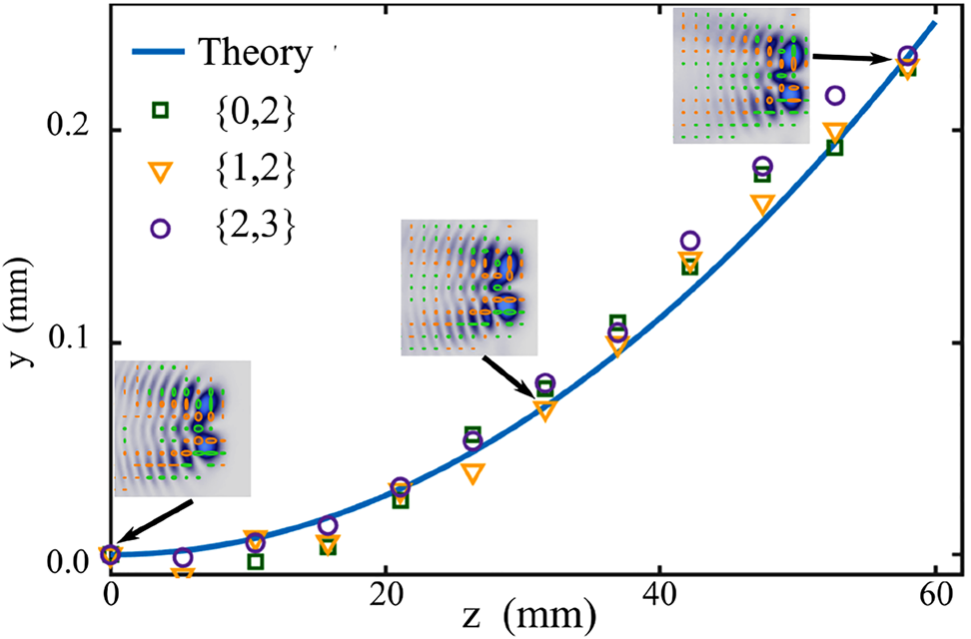
Shift of the *y*-coordinate of the maximum intensity lobe position for three AVWs upon free-space propagation. The continuous curve represents the expected position predicted by theory, whereas the data points correspond to experiment for the cases **AVW**_0,2_(*η*, *ξ*, *z*) (squares), **AVW**_1,2_(*η*, *ξ*, *z*) (triangles) and **AVW**_2,3_(*η*, *ξ*, *z*) (circles). Notice that all three cases accelerate in an identical way. Insets show the transverse polarisation distribution overlapped with the intensity profile of the **AVW**_1,2_(*η*, *ξ*, *z*) at three different planes.

The vector modes described by Eq. (3) and shown in Figure 2 propagate along parabolic trajectories maintaining not only their intensity and polarisation distribution but also a maximum coupling between both. We corroborated this by tracking the transverse spatial coordinates (*x*, *y*) of one of the lobes of maximum intensity as a function of their propagation distance *z*. We observed that while the *x* coordinate remains almost constant, the *y* coordinate shifts following a quadratic trend. Figure 3, in which the coordinate *y* is plotted against the propagation distance *z*, clearly shows such behaviour for a representative set of AVWs given by **AVW**_0,2_(*η*, *ξ*, *z*), **AVW**_1,2_(*η*, *ξ*, *z*) and **AVW**_2,3_(*η*, *ξ*, *z*). Since all modes shown were generated with the same initial parameters *k* and *κ*, they accelerated in an identical way. Insets show examples of the polarisation distribution overlapped with the intensity distribution at three propagation distances *z* = 0 mm, *z* = 20 mm and *z* = 60 mm for the mode **AVW**_1,2_(*η*, *ξ*, *z*).

**Figure 4:**
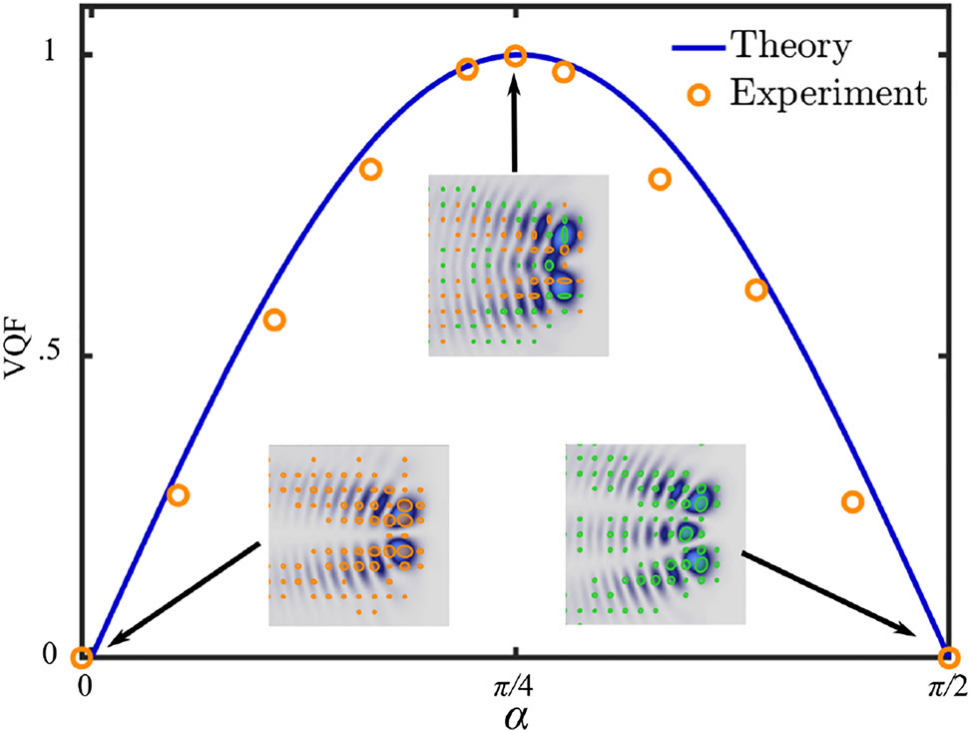
Vector quality factor (VQF) as a function of the weighing coefficient *α* ∈ [0, *π*/2] for the mode **AVW**_2,3_(*η*, *ξ*, *z* = 0). Insets show the intensity and polarisation distributions for *α* = 0, *π*/4 and *π*/2.

As mentioned earlier, AVWs can be generated with arbitrary degrees of non-separability, evolving from scalar to vector, via the parameter *α* (see Eq. (3)). More precisely, as *α* increases from 0 to *π*/4 the mode changes monotonically from a pure scalar mode with RHCP (*α* = 0) to a pure scalar mode with LHCP (*α* = *π*/2), passing through a pure vector mode (*α* = *π*/4). Intermediate values of *α* produce vector modes with intermediate degrees of non-separability, which can be measured through the concurrence or vector quality factor (VQF), which is a measure borrowed from quantum mechanics that allows to quantify the degree of coupling between the spatial and polarisation DoF [41–43]. Experimentally, the VQF can be quantified directly from the Stokes parameters as [44, 45],

(6) $$\text{VQF}=\sqrt{1-{\left(\frac{S_{1}}{S_{0}}\right)}^{2}-{\left(\frac{S_{2}}{S_{0}}\right)}^{2}-{\left(\frac{S_{3}}{S_{0}}\right)}^{2}},$$

where $S_{i}$ (*i* = 0, 1, 2, 3) is a number that results from integrating the Stokes parameters *S*_*i*_ over the entire transverse profile, i.e., $S_{i}{=\iint }_{-\infty }^{\infty }S_{i}\text{d}A$ . Figure 4 shows a representative example of the VQF as function of *α* for the specific case **AVW**_2,3_(*η*, *ξ*, *z* = 0). As expected, the VQF increases from 0 to 1, as *α* increases from 0 to *π*/4 and then it decreases back to zero, as *α* reaches the value *π*/2. Insets show the intensity profile overlapped with the polarisation distribution for three key values, namely *α* = 0, *π*/4 and *π*/2.

**Figure 5:**
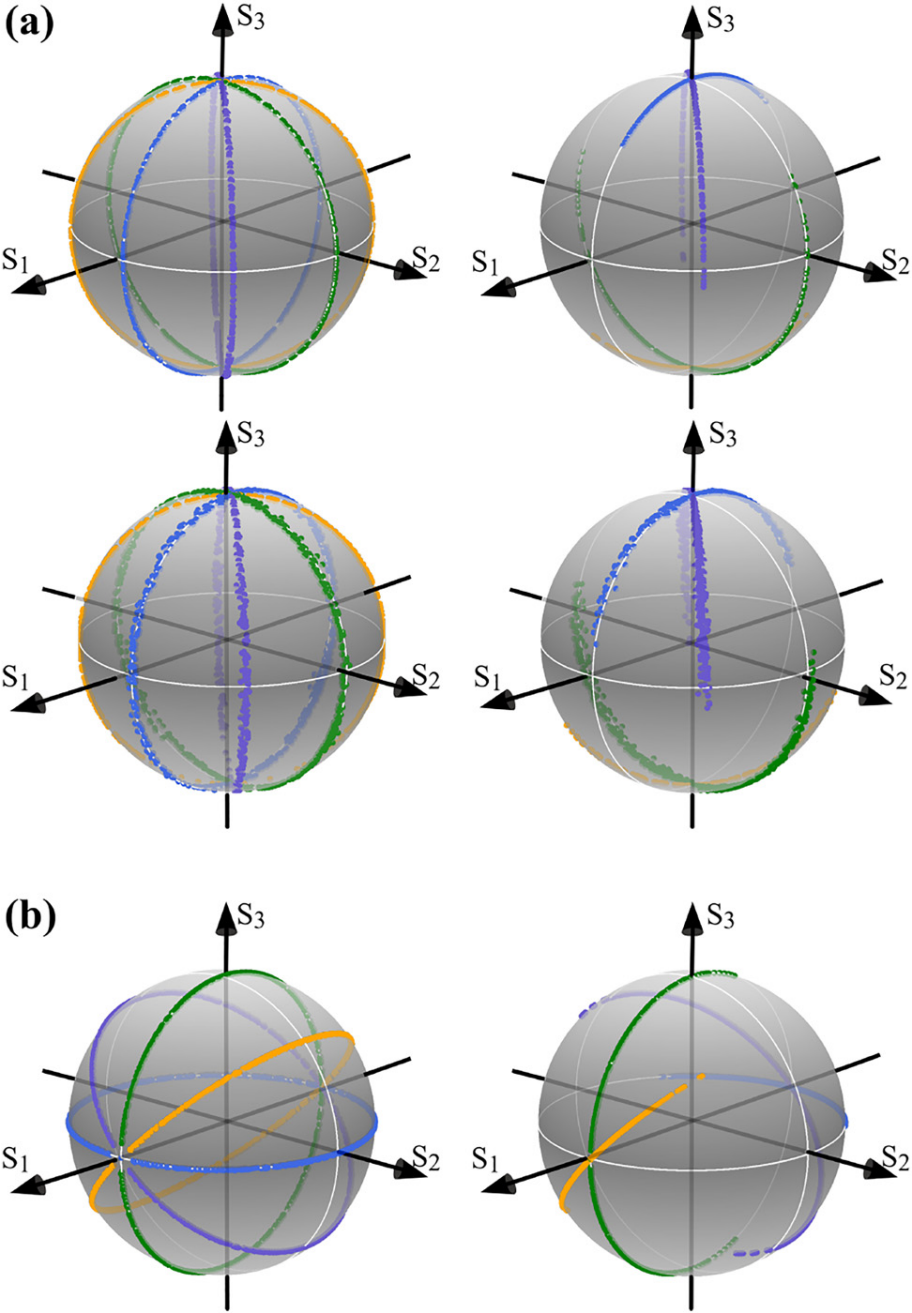
Geometric representation on the Poincaré sphere of the transverse polarisation distribution of the vector mode **AVW**_0,2_(*η*, *ξ*, *z* = 0) for (a) circular (numerical simulations on top and experiment on the bottom) and (b) horizontal/vertical polarisation basis. The left column shows the case of increasing values of the inter-modal phase, namely *β* = 0 (blue), *β* = *π*/4 (purple), *β* = *π*/2 (green) and *β* = 3*π*/4 (yellow). The right column shows modes with different weighting coefficients, namely *α* = *π*/12, *π*/6, *π*/4 and *π*/3, with the same inter-modal phases as in the left column.

Finally, we analyse the polarisation distribution of AVWs on the Poincaré sphere, for both circular and linear polarisation basis. For the case of circular polarisation basis, the first thing to notice is that any AVW contains all polarisation states, from linear to circular, nonetheless, all are oriented at exactly the same polarisation angle, differing only in their ellipticity. On the Poincaré sphere representation, all these polarisation states are mapped onto a great circle that intersects the North and South poles. Interestingly, a change in the inter-modal phase *β* originates all polarisation states to rotate in the same proportion. On the Poincaré sphere representation, this is seen as a rotation of the great circle around the *S*_3_ axis. The first row of Figure 5a, numerical simulation on top and experiment on the bottom, exemplifies this behaviour for the specific case **AVW**_0,2_(*η*, *ξ*, *z* = 0), with *β* = *π*/4 (purple), *β* = *π*/2 (green) and *β* = 3*π*/4 (yellow), as representative values. In addition, a change in the weighting coefficient *α* reduces the amount of right- (*α* < *π*/4) or left-handed (*α* > *π*/4) elliptical polarisation states in proportion to its value. On the Poincaré sphere representation, this behaviour is translated as incomplete circles or semi-circles, whose arc length is proportional to *α*. This behaviour is schematically represented on the right column of Figure 5a, numerical simulation on top and experiment on the bottom. For this example, we used the values *α* = *π*/12 (blue), *π*/6 (purple), *π*/4 (yellow) and *π*/3 (green) but for better visualisation of the change in length of each semicircle, we also used the same inter-modal phases as in the previous case, which as we know only rotates the circles around the *S*_3_ axis. Notice that as *α* increases from 0 to *π*/4, the semicircles increase in length from the north to the south pole and decrease in a similar way in the interval *α* ∈ [*π*/4, *π*/2]. Similar behaviour occurs in the case of horizontal/vertical linear polarisation basis, the main difference being, the great circles intersect the cross points between the *S*_1_ axis of the sphere, and rotate around the same axis when changing *β*. This behaviour is illustrated through numerical simulations in the left panel of Figure 5b. The effect of *α* is as in the circular basis, as also illustrated in the right panel of Figure 5b.

In summary, we have introduced theoretically and demonstrated experimentally a new family of vector beams with the ability to accelerate along parabolic trajectories upon free space propagation. Such accelerating beams differ quite dramatically from common vector beams, which always propagate along straight trajectories. These families of vector beams are constructed as a weighted superposition of the spatial and polarisation DoF carrying an inter-modal phase. To generate them, the spatial DoF is encoded in a set of orthogonal solutions of the 1D Schrödinger equations with a quartic potential, known as accelerating waves. An important feature of such modes is their propagation-invariant spatial and polarisation structures, as we corroborated experimentally. Further, the weighting coefficient allows tuning from purely scalar to completely vectorial, passing through intermediate states, which was also corroborated experimentally using the well-known measure of concurrence from quantum mechanics adapted for vector beams. Another important feature of these accelerating vector modes lies in their transverse polarisation distribution, which is mapped onto great circles on the Poincaré sphere. In particular, in the circular polarisation basis, the great circles intersect the North and South poles and contain states of polarisation from linear to circular, all with the same ellipticity angle. For comparison, cylindrical vector modes are mapped to the equator, a great circle or to the whole Poincaré sphere (known as full-Poincaré modes), depending on the spatial mode and polarisation basis [28, 46, 47]. Noteworthy, the inter-modal phase allows rotating the circle of polarisation around the Poincaré sphere, leaving the points on the North and South poles fixed. Given their interesting properties, we expect AVWs to find applications in fields such as optical manipulation, laser material processing, among others. As a final comment, our AVWs present several advantages over previously demonstrated Airy vector beams, both, from the fundamental and from the applications aspect. From the fundamental aspect, AVWs feature a more rich polarisation structure, which can be tuned from a completely scalar mode to a completely vectorial one. Furthermore, our AVWs retain their polarisation structure upon propagation, which does not happen with airy vector beams, since, as it has been demonstrated, upon propagation the vortex beam does not follow the same parabolic trajectory as the Airy beam [48, 49]. From the application side, it has become topical to use the polarisation DoF to control the polarisation of focussed vector beams, either for applications in optical tweezers or in laser material processing [15, 27]. Hence AVWs with richer polarisation structures suits better than Airy vector beams with polarisation structures restricted to cylindrical polarisation inherited from the cylindrical vector modes.

## References

[1] Siviloglou G. A., Christodoulides D. N. (2007). Accelerating finite energy Airy beams. Opt. Lett..

[2] Siviloglou G. A., Broky J., Dogariu A., Christodoulides D. N. (2007). Observation of accelerating airy beams. Phys. Rev. Lett..

[3] Efremidis N. K., Chen Z., Segev M., Christodoulides D. N. (2019). Airy beams and accelerating waves: an overview of recent advances. Optica.

[4] Bandres M. A. (2008). Accelerating parabolic beams. Opt. Lett..

[5] Bandres M. A. (2009). Accelerating beams. Opt. Lett..

[6] Greenfield E., Segev M., Walasik W., Raz O. (2011). Accelerating light beams along arbitrary convex trajectories. Phys. Rev. Lett..

[7] Zhang P., Hu Y., Li T. (2012). Nonparaxial Mathieu and Weber accelerating beams. Phys. Rev. Lett..

[8] Rosales-Guzmán C., Mazilu M., Baumgartl J., Rodríguez-Fajardo V., Ramos-García R., Dholakia K. (2013). Collision of propagating vortices embedded within Airy beams. J. Opt..

[9] Ruelas A., Davis J. A., Moreno I., Cottrell D. M., Bandres M. A. (2014). Accelerating light beams with arbitrarily transverse shapes. Opt. Express.

[10] Patsyk A., Bandres M. A., Bekenstein R., Segev M. (2018). Observation of accelerating wave packets in curved space. Phys. Rev. X.

[11] Aleahmad P., Miri M.-A., Mills M. S., Kaminer I., Segev M., Christodoulides D. N. (2012). Fully vectorial accelerating diffraction-free Helmholtz beams. Phys. Rev. Lett..

[12] Davis J. A., Mitry M. J., Bandres M. A., Ruiz I., McAuley K. P., Cottrell D. M. (2009). Generation of accelerating Airy and accelerating parabolic beams using phase-only patterns. Appl. Opt..

[13] Forbes A., de Oliveira M., Dennis M. R. (2021). Structured light. Nat. Photonics.

[14] Rubinsztein-Dunlop H., Forbes A., Berry M. V. (2017). Roadmap on structured light. J. Opt..

[15] Rosales-Guzmán C., Ndagano B., Forbes A. (2018). A review of complex vector light fields and their applications. J. Opt..

[16] Konrad T., Forbes A. (2019). Quantum mechanics and classical light. Contemp. Phys..

[17] Eberly J. H., Qian X.-F., Al Qasimi A. (2016). Quantum and classical optics–emerging links. Phys. Scripta.

[18] Toninelli E., Ndagano B., Vallés A. (2019). Concepts in quantum state tomography and classical implementation with intense light: a tutorial. Adv. Opt. Photon..

[19] Forbes A., Aiello A., Ndagano B. (2019). Classically entangled light. *Progress in Optics*.

[20] Töppel F., Aiello A., Marquardt C., Giacobino E., Leuchs G. (2014). Classical entanglement in polarization metrology. New J. Phys..

[21] Hu X.-B., Zhao B., Zhu Z.-H., Gao W., Rosales-Guzmán C. (2019). In situ detection of a cooperative target’s longitudinal and angular speed using structured light. Opt. Lett..

[22] Berg-Johansen S., Töppel F., Stiller B. (2015). Classically entangled optical beams for high-speed kinematic sensing. Optica.

[23] Ndagano B., Nape I., Cox M. A., Rosales-Guzmán C., Forbes A. (2018). Creation and detection of vector vortex modes for classical and quantum communication. J. Lightwave Technol..

[24] Ndagano B., Perez-Garcia B., Roux F. S. (2017). Characterizing quantum channels with non-separable states of classical light. Nat. Phys..

[25] Otte E., Denz C. (2020). Optical trapping gets structure: structured light for advanced optical manipulation. Appl. Phys. Rev..

[26] Sit A., Bouchard F., Fickler R. (2017). High-dimensional intracity quantum cryptography with structured photons. Optica.

[27] Yang Y., Ren Y., Chen M., Arita Y., Rosales-Guzmán C. Optical trapping with structured light: a review. Adv. Phot..

[28] Zhan Q. (2009). Cylindrical vector beams: from mathematical concepts to applications. Adv. Opt. Photon..

[29] Dudley A., Li Y., Mhlanga T., Escuti M., Forbes A. (2013). Generating and measuring nondiffracting vector Bessel beams. Opt. Lett..

[30] Otte E., Denz C. (2018). Sculpting complex polarization singularity networks. Opt. Lett..

[31] Yao-Li, Hu X.-B., Perez-Garcia B. (2020). Classically entangled Ince–Gaussian modes. Appl. Phys. Lett..

[32] Rosales-Guzmán C., Hu X.-B., Rodríguez-Fajardo V., Hernandez-Aranda R. I., Forbes A., Perez-Garcia B. (2021). Experimental generation of helical Mathieu–gauss vector modes. J. Opt..

[33] Singh K., Buono W. T., Forbes A., Dudley A. (2021). Accelerating polarization structures in vectorial fields. Opt. Express.

[34] Hu X.-B., Perez-Garcia B., Rodríguez-Fajardo V., Hernandez-Aranda R. I., Forbes A., Rosales-Guzmán C. (2021). Free-space local nonseparability dynamics of vector modes. Photon. Res..

[35] Zhou J., Liu Y., Ke Y., Luo H., Wen S. (2015). Generation of Airy vortex and Airy vector beams based on the modulation of dynamic and geometric phases. Opt. Lett..

[36] Davis J. A., Mitry M. J., Bandres M. A., Cottrell D. M. (2008). Observation of accelerating parabolic beams. Opt. Express.

[37] Banerjee K., Bhatnagar S. P., Choudhry V., Kanwal S. S., Robert Bates D. (1978). The anharmonic oscillator. Proc. R. Soc. A: Math. Phys. Eng. Sci..

[38] Driscoll T. A., Hale N., Trefethen L. N. (2014). Chebfun Guide.

[39] Rosales-Guzmán C., Hu X.-B., Selyem A. (2020). Polarisation-insensitive generation of complex vector modes from a digital micromirror device. Sci. Rep..

[40] Scholes S., Kara R., Pinnell J., Rodríguez-Fajardo V., Forbes A. (2019). Structured light with digital micromirror devices: a guide to best practice. Opt. Eng..

[41] McLaren M., Konrad T., Forbes A. (2015). Measuring the nonseparability of vector vortex beams. Phys. Rev. A.

[42] Ndagano B., Brüning R., McLaren M., Duparré M., Forbes A. (2015). Fiber propagation of vector modes. Opt. Express.

[43] Zhao B., Hu X.-B., Rodríguez-Fajardo V. (2019). Real-time Stokes polarimetry using a digital micromirror device. Opt. Express.

[44] Adam S., Rosales-Guzmán C., Croke S., Forbes A., Franke-Arnold S. (2019). Basis-independent tomography and nonseparability witnesses of pure complex vectorial light fields by Stokes projections. Phys. Rev. A.

[45] Manthalkar A., Nape I., Bordbar N. T. (2020). All-digital Stokes polarimetry with a digital micromirror device. Opt. Lett..

[46] Maurer C., Jesacher A., Fürhapter S., Bernet S., Ritsch-Marte M. (2007). Tailoring of arbitrary optical vector beams. New J. Phys..

[47] Galvez E. (2012). Vector beams in free space. The Angular Momentum of Light.

[48] Wei B.-Y., Liu S., Chen P. (2018). Vortex airy beams directly generated via liquid crystal q-airy-plates. Appl. Phys. Lett..

[49] Dai H. T., Liu Y. J., Luo D., Sun X. W. (2011). Propagation properties of an optical vortex carried by an airy beam: experimental implementation. Opt. Lett..

